# Supplementary material for: Natural Methoxyphenol Compounds: Antimicrobial Activity against Foodborne Pathogens and Food Spoilage Bacteria, and Role in Antioxidant Processes
Source: Foods. 2021 Aug 5;10(8):1807. doi: 10.3390/foods10081807 (PMC8392586; doi:10.3390/foods10081807)
Supplement: Supplementary file 1 [file foods-10-01807-s001.zip › foods-1301181-supplementary.pdf]

foods

Article

Natural methoxyphenol compounds: antimicrobial activity against food-borne pathogens and -spoilage bacteria, and role in the antioxidant processes

Elena Orlo, Chiara Russo*, Roberta Nugnes, Margherita Lavorgna, Marina Isidori

Department of Environmental, Biological and Pharmaceutical Sciences and Technologies, University of Campania "Luigi Vanvitelli", Via Vivaldi 43, 81100 Caserta, Italy;
elena.orlo@unicampania.it (E.O.); roberta.nugnes@unicampania.it (R.N.); margherita.lavorgna@unicampania.it (M.L.); marina.isidori@unicampania.it (M.I.)
* Correspondence: chiara.russo@unicampania.it

Supplementary Material

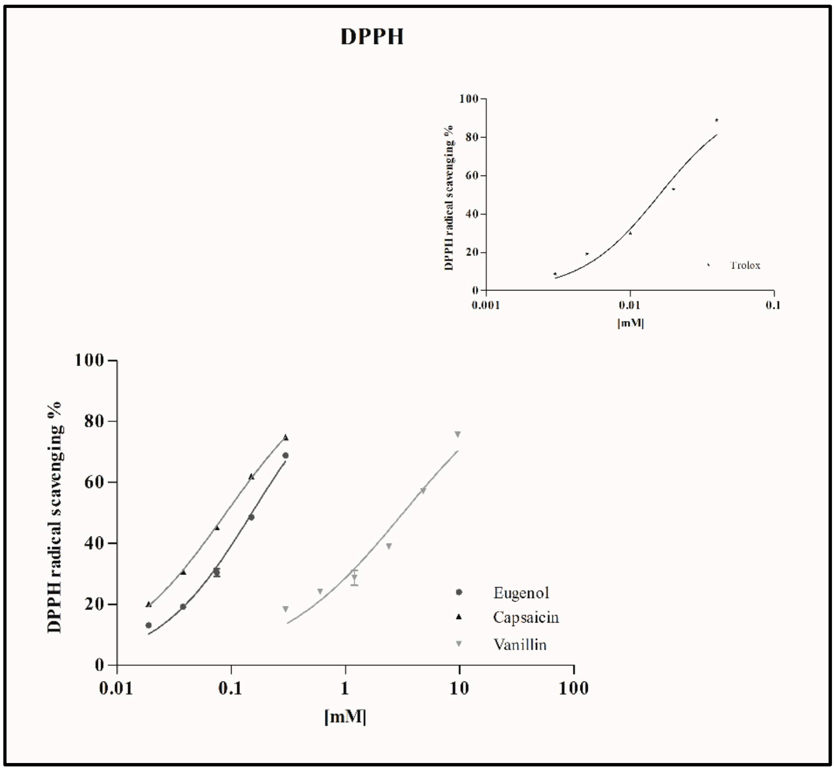

Figure S1. Concentration-effect curves in DPPH assay

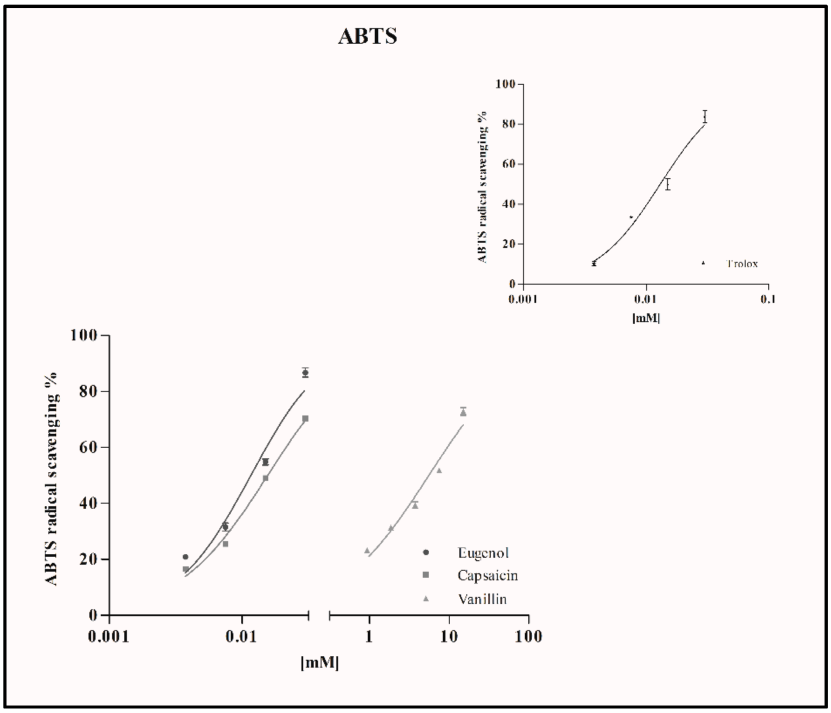

Figure S2. Concentration-effect curves in ABTS assay

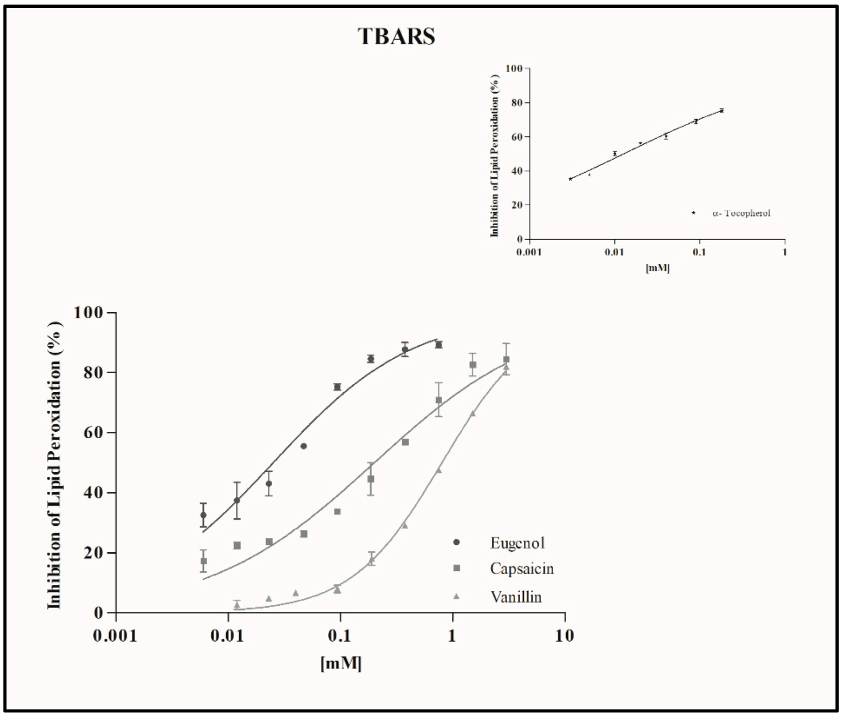

Figure S3. Concentration-effect curves in TBARS assay

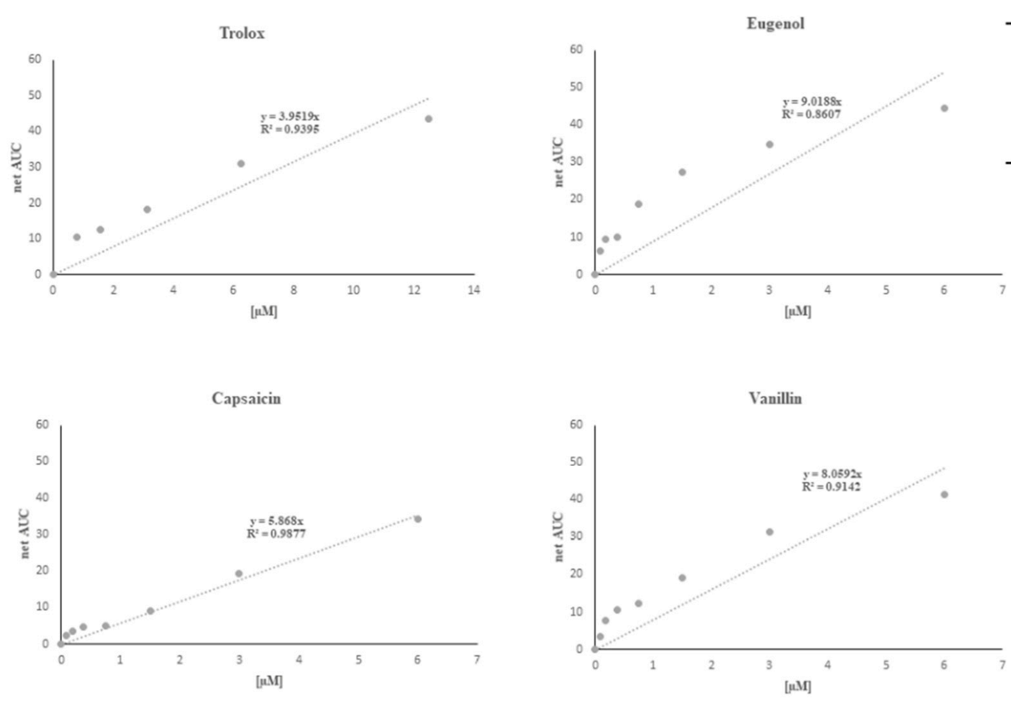

	m
**Trolox**	**4.05 ± 0.13**
**Eugenol**	8.60 ± 0.59[a*]
**Capsaicin**	6.44 ± 0.78[a]
**Vanillin**	7.34 ± 0.01[a*]

Figure S4. Calibration curves representative of one of the three experiments carried out in ORAC assay.
m value is the mean value coming from three independent experiments ± Standard deviation. Asterisks highlight significant differences from Trolox (One Way-ANOVA, Dunnett's test: *p<0.05). Different letters for p<0.05 (One Way-ANOVA, Tukey's Multiple Comparison Test).

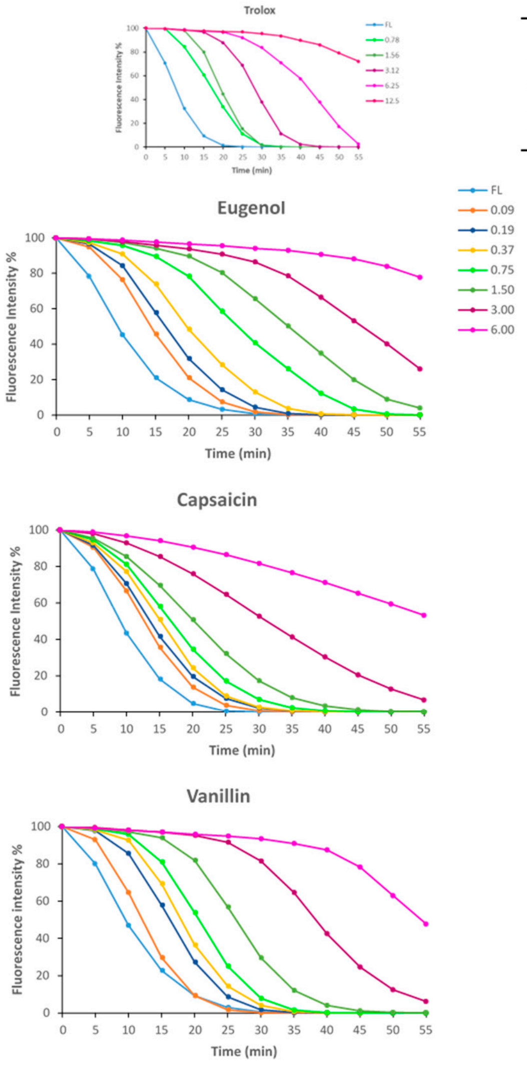

	max AUCnet
**Trolox**	**31.99 ± 1.22**
**Eugenol**	42.88 ± 2.18[a*]
**Capsaicin**	36.41 ± 3.24[a]
**Vanillin**	39.12 ± 3.30[a]

Figure S5. Fluorescence decay curves. Kinetic profiles of fluorescence consumption mediated by AAPH de-rived peroxyl radicals in absence (only fluorescein, FL) or in presence of trolox or eugenol, cap-saicin and vanillin. With increasing concentrations (μM) of Trolox or phytochem-icals, the time needed for quenching of FL and, consequently, the net area under the curve (netAUC) increased. maxAUCnet values of Trolox (at 6.25 μM) and phytochemicals (at 6.00 μM) with standard deviation. Asterisks highlight significant differences from Trolox (One Way-ANOVA, Dunnett's test: *p<0.05). Different letters for p<0.05 (One Way-ANOVA, Tukey's Multiple Comparison Test).

The area under the fluorescence decay curve (AUC) was calculated as:

AUC = (0.5 + F5/F0 + F10/F0 + F15/F0 + …… + Fn/F0) x 5

where F0= fluorescence reading at time 0; Fn= fluorescence reading at time n.

From the AUC of the blank and AUC of phytochemicals, net AUC was calculated using the equation:

AUCnet= AUCsample – AUCblank

Table S1. Chromatic table. Chromatic table summarizing antibacterial activity exerted by phytomolecules in different bacterial strains. Gray scale was used for the significant increase in sensitivity of bacterial strains (darkest gray for the highest sensitivity) to eugenol, capsaicin and vanillin. The increasing number of symbols was used to identify the significant increase in antibacterial activity exerted by phytochemicals (three symbols for the highest effectiveness). Significance was considered for p<0.05 (Two Way ANOVA- Bonferroni posttests).

Bacterial strain	IC_{50}		
	Eugenol	Capsaicin	Vanillin
E. coli ●	●●●	●●●	●●●
P. aeruginosa ♦	♦♦♦	♦♦♦	♦♦
S. putrefaciens ■	■■■	■■■	■■■
S. aureus ✱	✱✱✱	✱✱✱	✱✱✱
L. plantarum x	x x	-	x
B. thermosphacta ▲	▲▲▲	▲▲▲ ▲▲	▲▲

- not determined

Table S2. Chromatic table. Chromatic table summarizing antioxidant activity exerted by phytomolecules using different assays. Gray scale was used for the significant increase in sensitivity/suitability of antioxidant assays (darkest gray for the highest sensitivity) to test eugenol, capsaicin and vanillin. The increasing number of symbols was used to identify the significant increase in antioxidant activity exerted by phytochemicals (three symbols for the highest effectiveness). Significance was considered for p<0.05 (Two Way ANOVA- Bonferroni posttests).

Assay	IC_{50}		
	Eugenol	Capsaicin	Vanillin
DPPH ●	●●	●●	●
ABTS ♦	♦♦♦	♦♦♦	♦
TBARS ■	■■■	■■	■
ORAC ✱	✱✱✱	✱✱✱	✱✱✱
