# Supplementary figures and images for: Natural Methoxyphenol Compounds: Antimicrobial Activity against Foodborne Pathogens and Food Spoilage Bacteria, and Role in Antioxidant Processes
Source: Foods. 2021 Aug 5;10(8):1807. doi: 10.3390/foods10081807 (PMC8392586; doi:10.3390/foods10081807)

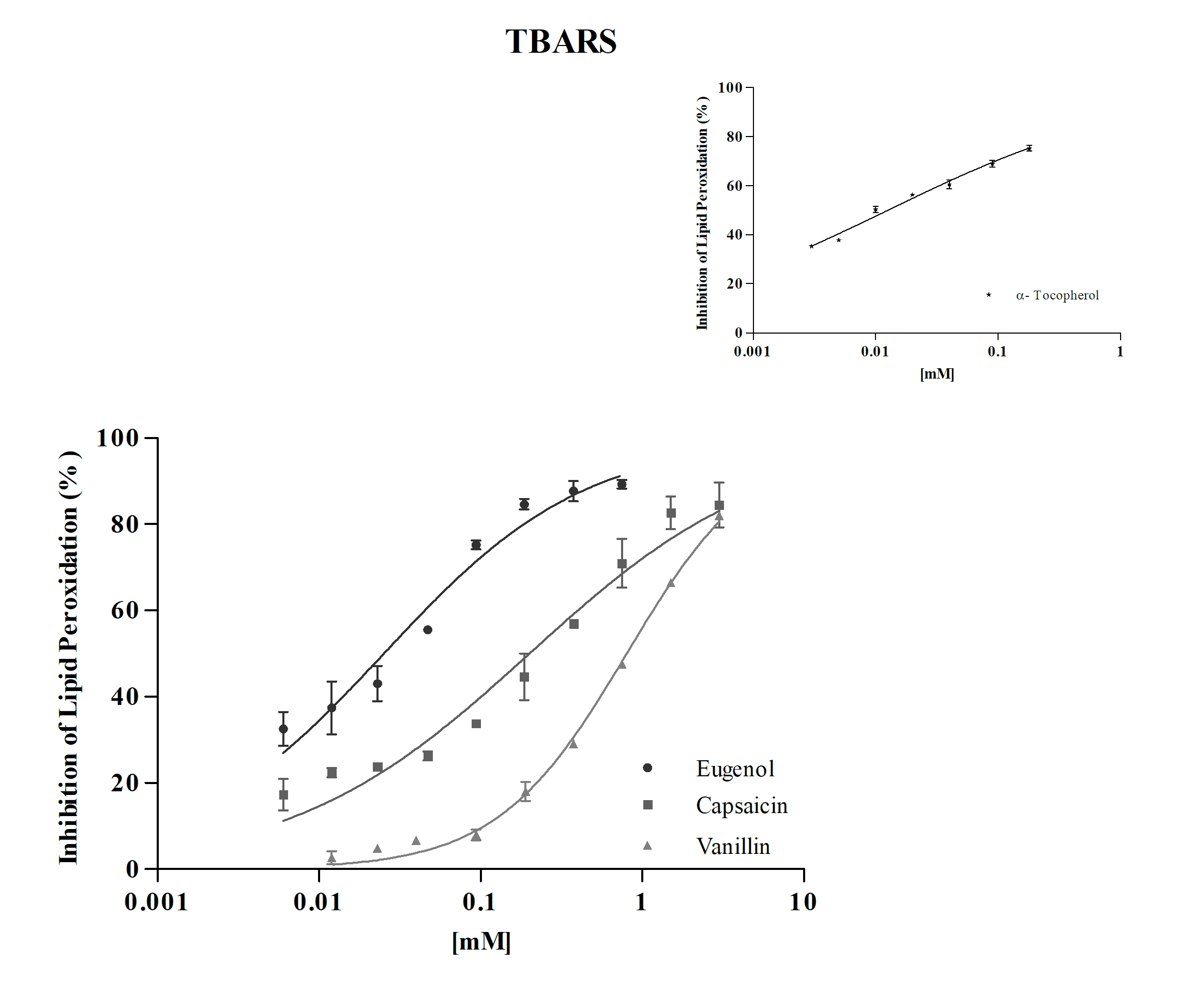

Supplement: Supplementary file 1 [file foods-10-01807-s001.zip › Figure S3.tif]

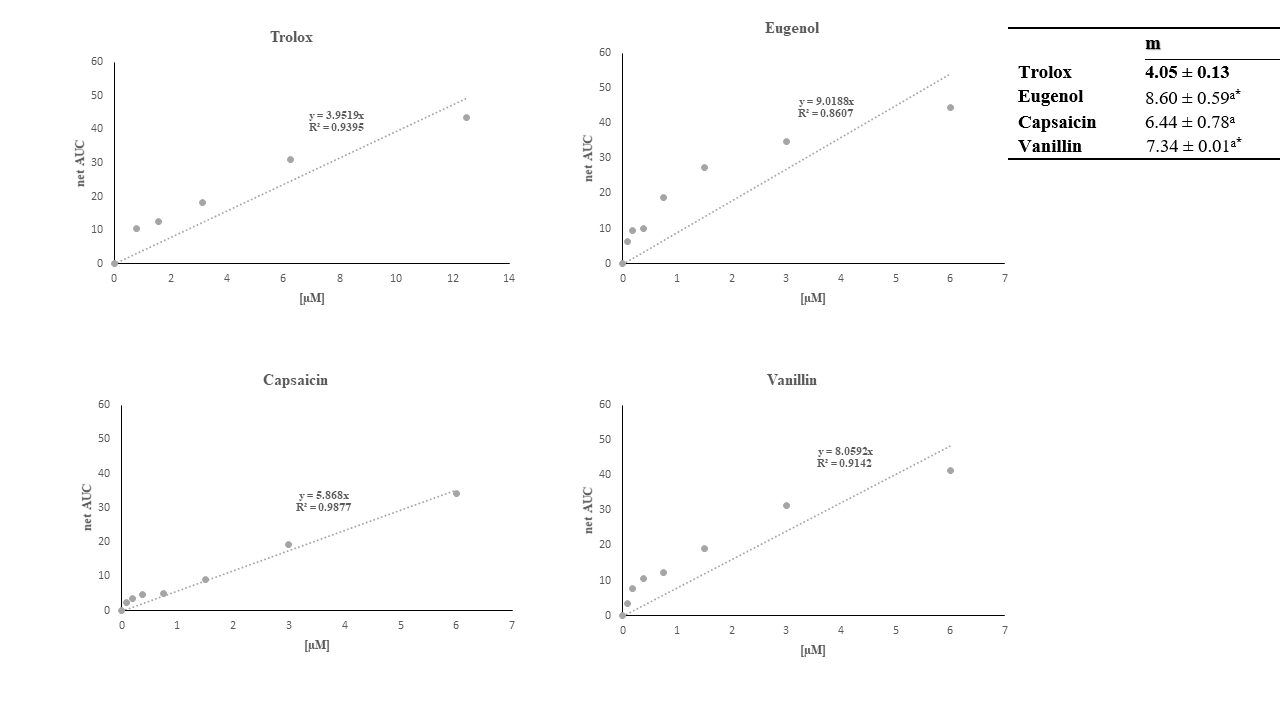

Supplement: Supplementary file 1 [file foods-10-01807-s001.zip › Figure S4.tif]

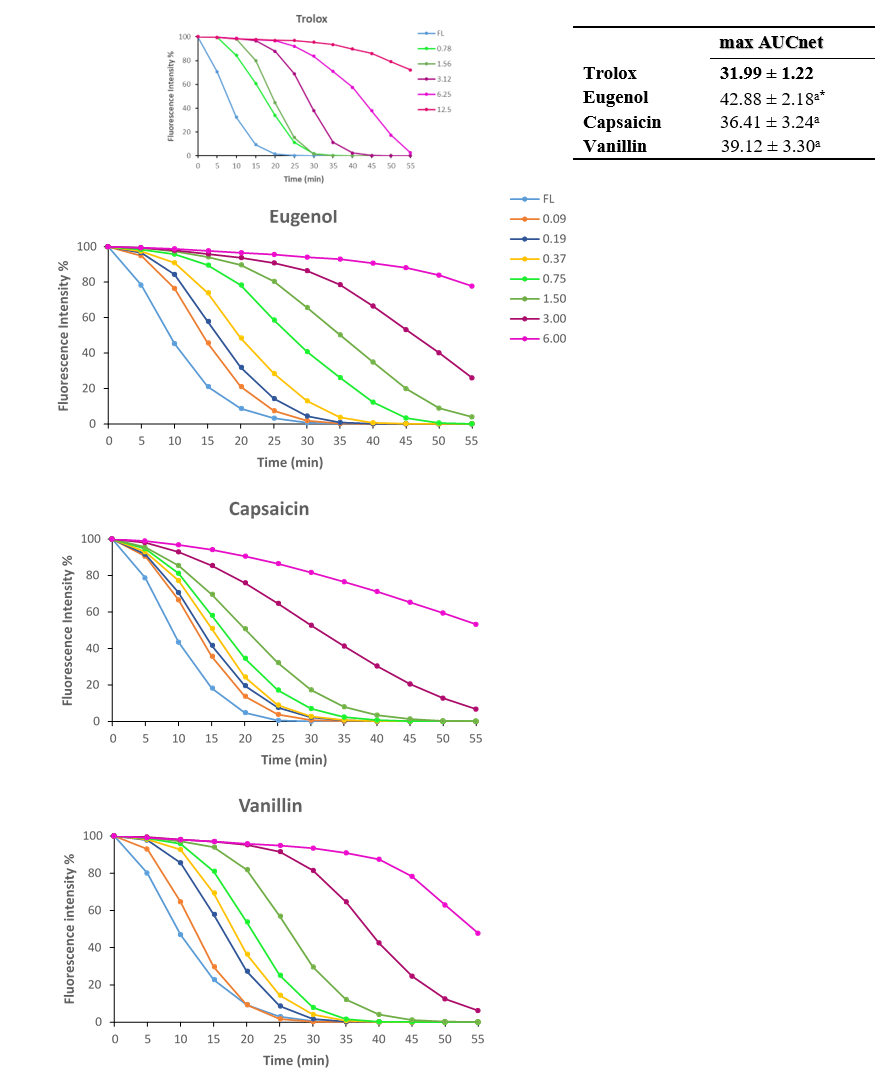

Supplement: Supplementary file 1 [file foods-10-01807-s001.zip › Figure S5.tif]

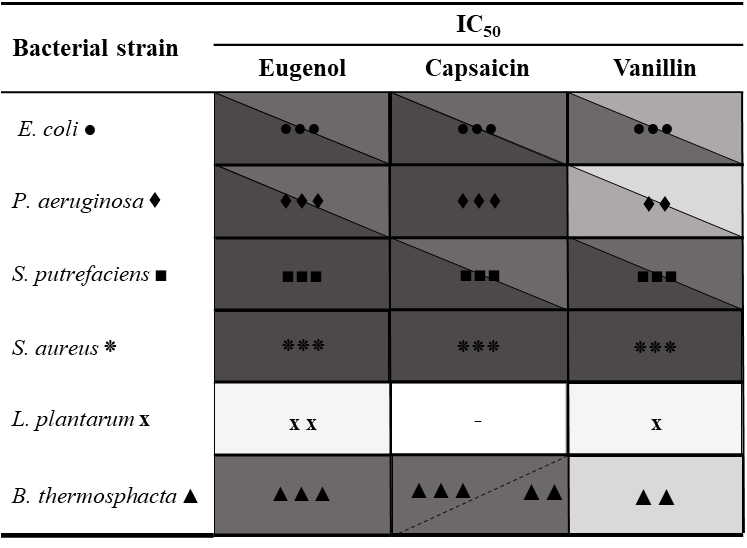

Supplement: Supplementary file 1 [file foods-10-01807-s001.zip › Table S1.tif]

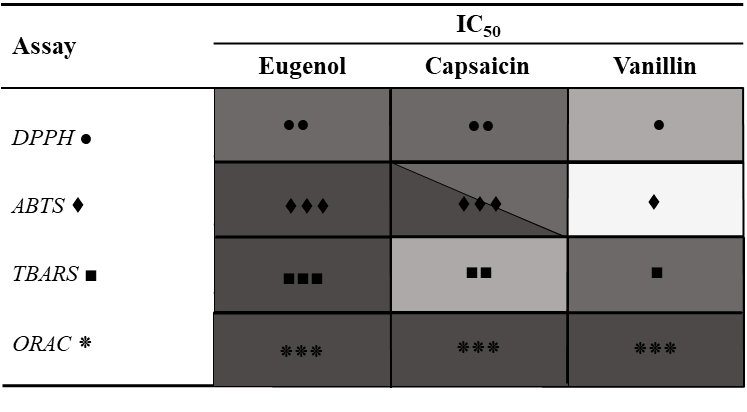

Supplement: Supplementary file 1 [file foods-10-01807-s001.zip › Table S2.tif]
